# Humic-Based Polyelectrolyte Complexes for Dust Suppression

**DOI:** 10.3390/polym15061514

**Published:** 2023-03-18

**Authors:** Alexander Volikov, Evgeniya A. Karpukhina, Konstantin S. Larionov, Daniil A. Kozlov, Irina V. Perminova

**Affiliations:** 1 Department of Chemistry, Lomonosov Moscow State University, Leninskie Gory 1-3, 119991 Moscow, Russia; 2 Kurnakov Institute of General and Inorganic Chemistry of RAS, Leninsky Prospect 31, 119991 Moscow, Russia

**Keywords:** dust suppressant, particulate matter, humic substances, road dust, wind erosion resistance, silsesquioxane, polyelectrolyte complexes

## Abstract

The present study proposes a novel application of humic substance–aminosilsesquioxane polyelectrolyte complexes (HS–ASQ) as dust suppressants. These complexes are synthesized through the reaction between humic substances (HS) and 3-aminopropyltriethoxysilane (APTES) in aqueous solution, resulting in the formation of active silanol groups that can bind to mineral surfaces and condense, forming gels. The HS–ASQ compositions were found to have a high sorption capacity for dust particles and could form coatings on their surface without cementing the dust, making them potentially useful for environmental applications. The viscosity of the HS–ASQ compositions can be controlled by adding carboxymethylcellulose (CMC), which also enhances their dust suppression abilities. Different compositions of HS–ASQ were synthesized by varying the proportions of APTES and CMC, and dust treated with these samples was assessed for its resistance to wind erosion using a laboratory-scale setup. Treatment with the HS–ASQ composition resulted in substantial reductions in PM10 and PM2.5 concentrations (particulate matter with aerodynamic diameters of 10 µm and 2.5 µm, respectively) of up to 77% and 85%, respectively, compared to the control.

## 1. Introduction

The World Health Organization estimates that air pollution causes more than seven million premature deaths every year [1]. Dust and typical air pollution, particularly in large cities, is a significant contributor to this problem. Particulate matter (PM) in the air, referred to as PM10 (coarse particles with a diameter ≤ 10 µm) and PM2.5 (small particles with a diameter ≤ 2.5 µm), can penetrate the lungs and blood, causing respiratory [2,3] and cardiovascular diseases [4], coronary heart disease [5], and even Alzheimer’s disease [6] when inhaled. Dust can also negatively impact the environment, affecting plant growth and containing harmful elements such as heavy metals and polyaromatic hydrocarbons [7]. For these reasons, in their new recommendations, the WHO reduced the safe average annual concentration level in the air of PM2.5 particles by half and PM10 particles by a quarter to 5 and 15 μg/m^3^, respectively [1].

According to data from the United States Environmental Protection Agency for 2014 [8], the main sources of dust included wildfires, non-road and on-road dust, industrial processes, and fuel combustion, where industrial processes contributed the most to pollution, at 63% and 87% for PM2.5 and PM10, respectively. Coal mining contributes highly to pollution; therefore, much of the recent work has been devoted to creating effective dust suppressors for coal particles [9,10,11,12,13,14,15,16,17]. If we consider dust sources in cities far from industrial centers and do not consider wildfires, then the main source of dust is road dust, at 48% and 62% for PM2.5 and PM10, respectively [8]. State transportation departments and local agencies employ various maintenance techniques, such as paving and the use of dust suppressants, to reduce the negative impacts of fugitive dust on human health and safety [18]. Dust suppressants are the most widely used method because of their ease of use and low cost. They are usually applied by watering roads and soils with sprinklers to transfer dust particles to the water phase. Plain water is usually used in this method, but it evaporates quickly and dusty particles can become subject to wind erosion once again [19]; therefore, the use of various additives, such as dust suppressants, is much more efficient.

In the review in [20], dedicated to unpaved road dust suppressors, the authors classified them into three types according to their composition: (1) organic compound-based, (2) biopolymer and chemical combination, and (3) inorganic compound-based dust suppressants. Considering inorganic dust suppressors, one of the most simplest are chloride salts (CaCl_2_ and MgCl_2_) [21,22,23]. They possess a common mechanism for controlling dust on the basis of their hygroscopic properties, allowing them to attract moisture from the air and bind particles together on the aggregate surface. Their main advantage is their low cost and relatively high efficiency, reaching 50–70% dust suppression, as shown in several works [21,23]. However, the authors of [22] demonstrated their low efficiency in the Mediterranean urban area; they do not have a long-term effect and require occasional use on the road [24]. Organic compound-based dust suppressants are more active and have a longer effect, among them, the use of liquid polymers is notable. Lee et al. [25] mixed liquid amphiphilic poly(ethylene oxide-b-propylene oxide-b-ethylene oxide) (PEO–PPO–PEO) triblock copolymer and liquid hydrophilic polyethylene glycol (PEG) in deionized water to create a liquid polymer solution. The results of laboratory tests showed that a 7% PEG solution reduced PM10 by 87% and PM2.5 by 86%, while 3–5% of the PEO-PPO-PEO solution reduced PM10 by 91–89% and PM2.5 by 89%. Despite the high merits of these compositions, there is a risk of secondary contamination due to decomposition into their low-molecular-weight components. Using biopolymer dust suppressants has no disadvantage, as they are easily immersed in the environment.

Biopolymers, such as guar and xanthan gum, can be used as water additives to suppress dust and increase surface strength and ductility by bonding with soil particles [26]. This reduces external damage and provides a long-term dust suppression effect [27]. Starch is also a popular biopolymer, Dang et al. [28] used oxidized corn starch, gelatin, and sodium carboxymethylcellulose to create dust suppressants, which demonstrated 68% and 79% PM2.5 and PM10 fugitive dust particles suppressions, respectively. Zhu et al. [29] proposed a composition based on potato starch and bentonite that removed 89% of PM2.5 and 94% of PM10 in a laboratory-scale experiment.

**Figure 1:**
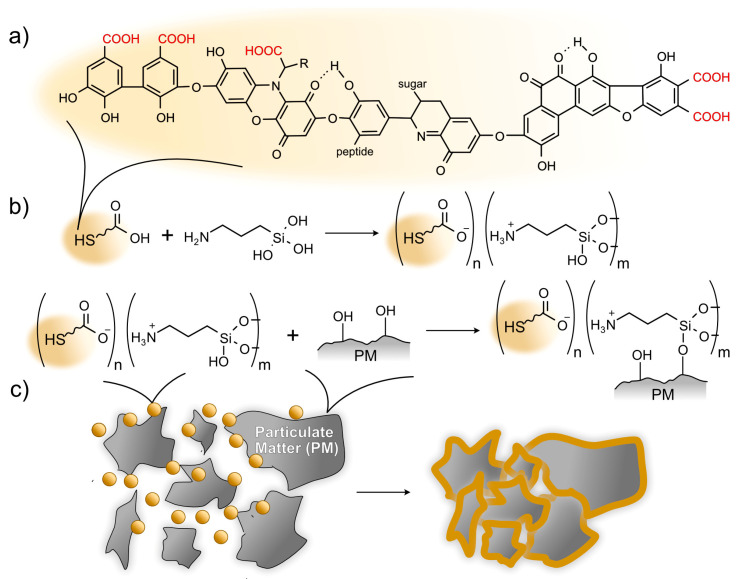
Model structure of humic acids [31]. (**a**) Scheme of the interaction of humic substances and 3-aminopropyltrihydroxysilane with the formation of humic substance–aminosilsesquioxane polyelectrolyte complexes (HS–ASQ) (**b**) and their binding to particulate matter (**c**).

Among natural biopolymers, there are humic substances (HSs), which can be considered as dust suppressants. HSs are formed by the oxidative degradation of biomass and have high structural heterogeneity and stability [30]. One of the hypothetical structures of HS [31] is shown in Figure 1a. Cotosa et al. [32] used molasses stillage with a 15% content of humic substances for dust suppression in a field experiment, showing a high efficiency. In another work [12], the authors used humic substances to modify grafted acrylamide to create an effective dust suppressant for coal mining. The carboxyl groups in the composition of the HS lead to a negatively charged substance. Thus, the HS could form polyelectrolyte complexes with positively charged polyelectrolytes such as chitosan [33]. We have previously proposed the synthesis of humic substance–aminosilsesquioxane polyelectrolyte complexes (HS–ASQ), formed due to the interaction of HS with 3-aminopropyltriethoxysilane (APTES) in a water solution [34]. The proposed synthetic scheme is shown in Figure 1b. As a result of APTES’s hydrolysis in water, positively charged aminosilsesquioxanes are formed because of the presence of amino groups in their composition, which form polyelectrolyte complexes with negatively charged HSs. Due to the formation of covalent siloxane bonds, HS–ASQ can interact with hydroxyl-containing surfaces and bind to them by mechanical inclusion in the gel formed during the condensation of HS–ASQ [34]. The same principle can be used to bind dust particles, which are mostly composed of mineral particles [35] (Figure 1c). HS–ASQ does not exhibit toxicity and it has a beneficial effect on soils and crops; therefore, their application can support the environment [36,37]. This study considers the possibility of applying HS–ASQ to bind to road dust.

## 2. Materials and Methods

### 2.1. Materials

Commercially available potassium humate (Powhumus, Humintech, Germany) isolated from leonardite was used as a humic material (CHP). The compound 3-aminopropyltriethoxysilane (APTES) was purchased from Penta Ltd. (Moscow, Russia). Sodium carboxymethyl cellulose (CMC) with a 1.03 degree of substitution (FL9, Wuxi, China) was used for viscosity regulation. Dust samples were collected from a rural field road (55.09985° N, 37.06803° E) and then dried. A fraction with a particle size less than 100 µm was selected using metal sieves (Vibrotechnik, St. Petersburg, Russia). All aqueous solutions were prepared with deionised water using a Simplicity 185 system (Millipore, Merck KGaA, Darmstadt, Germany). The pH of solutions was measured using an Ecotest-2000 pH meter (Econix, Moscow, Russia) equipped with a universal glass electrode. Analytical grade reagents (NaOH and HCl) were used to adjust the pH of the samples.

### 2.2. Preparation of HS–ASQ

**Figure 2:**
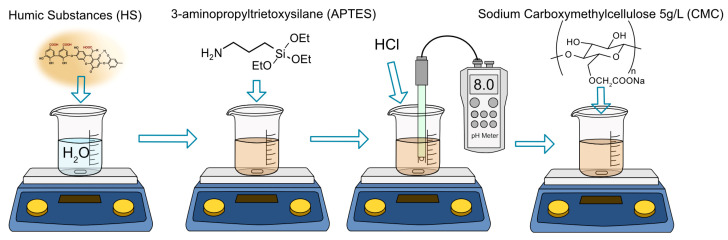
HS–ASQ preparation scheme.

The CHP sample was dissolved in water and used for preparation of CHP solutions at concentrations in the range from 2 up to 10 g/L. Then, APTES was added drop-wise to the CHP–CMC solutions upon continued stirring to yield the ratios of CHP:APTES in the range from 4:1 to 1:1 by weight. The pH of the CHP–APTES solution was adjusted to pH 8 using 1 M HCl. To enhance the viscosity of the CHP solution, a 5 g/L carboxymethylcellulose (CMC) solution in water was added to give resulting CHP:CMC mass ratios in the range from 8:1 to 2:1. The preparation scheme is presented in Figure 2. The prepared HS–ASQ complexes were denoted in this study as HS–ASQ:X CMC:Y, where X represents the mass percentage of APTES and Y represents the mass percentage of CMC in the synthesis process; for example, HS–ASQ:50 CMC:25 5 g/L means that the composition contains 5 g/L CHP, 2.5 g/L APTES, and 1.25 g/L CMC.

### 2.3. FTIR Instrumentation and Measurements

FTIR measurements were performed using a Vertex 70 FTIR spectrometer (Bruker Optik GmbH, Ettlingen, Germany) equipped with an ATR attachment with a diamond crystal. The spectra were recorded in the range of 4000–370 cm^–1^ with a resolution of 2 cm^–1^ and 64 scans. The crystal was heated to 40 °C. An amount of 5 μL of the solution was placed on the crystal, the drop was dried for 6–10 min (until the water peaks disappeared), and the spectrum of the resulting film was recorded. After automatic exportation, the data were processed using OPUS software (Bruker Optik GmbH 2012, version 7.2.139.1294). All spectra were smoothed using 9 points. Further data processing was carried out using Microsoft Excel 2021 (Microsoft, Redmond, WA, USA).

### 2.4. Scanning Electron Microscopy

Scanning electron microscopy (SEM) images were obtained using an Amber GMH microscope (Tescan, Brno, Czech Republic) microscope operated at an accelerating voltage of 5 kV using a secondary electron detector.

### 2.5. Powder X-ray Diffraction

Powder X-ray diffraction (PXRD) patterns were acquired using a powder diffractometer with a rotating anode D/MAX 2500 PC (Rigaku, Tokyo, Japan) in the reflection geometry (Bragg–Brentano) with Cu Kα1,2 radiation and a graphite monochromator. XRD patterns were collected in the 5–80°2θ range with a 0.02° step size. Identification of the diffraction peaks was carried out using the ICDD database (PDF2, 2020 release).

### 2.6. Viscosity Measurement

Viscosity was determined using the Ostwald VPG-1 viscosimeter (Ekros-Analitika, Moscow, Russia). The apparatus consisted of a capillary tube with two points located at different heights. Measurement of the time required for fluid flow from the first point to the second was recorded and carried out three times. The viscosimeter was calibrated using water as a standard fluid with known viscosity, and then the kinematic viscosity of the samples was calculated using the following formula:

(1) $$\eta =\frac{\eta_{y}}{\eta_{0}}=\frac{d_{y}}{d_{0}}\times \frac{t_{y}}{t_{0}},$$

where $\eta_{y}$ is the kinematic viscosity of the sample, $\eta_{0}$ is the kinematic viscosity of water (0.893 mm^2^/s at 25 °C), $d_{y}$ is the density of the sample, $d_{0}$ is the density of water, $t_{y}$ is the measured time for the sample, and $t_{0}$ is the measured time for water.

This study determined the viscosity of diluted samples in water with concentrations of less than 1%. Given these low concentrations, the density change was considered negligible; thus, the density of water was used in all calculations.

### 2.7. Sorption of HS–ASQ

The sorption experiment was carried out at room temperature (25 °C). For the experiment, we used stock solutions of the compositions HS–ASQ:100, HS–ASQ:50, and HS–ASQ:25 by diluting them with distilled water to concentrations in the range of 0.05–1 g/L and placed in 15 mL test tubes. Then, 100 mg of dust was added to the tubes, and they were fixed onto an overhead shaker for 24 h until sorption equilibrium was established. The concentration was determined spectrophotometrically by measuring the optical density at 254 nm and using a calibration curve for the initial humic substances. The amount of sorbed HS–ASQ was determined using the following equation:

(2) $$q=\left(C_{0}-C_{w}\right)*V/m,$$

where *q* is the amount of HSHQ (mg) sorbed onto 1 g of dust, *C*_0_ and *C_w_* are the initial and equilibrium concentrations of HSHQ in solution (mg/L), respectively, *V* is the volume of the solution (mL), and *m* is the mass of dust (g).

### 2.8. Wind Erosion Resistance Test

**Figure 3:**
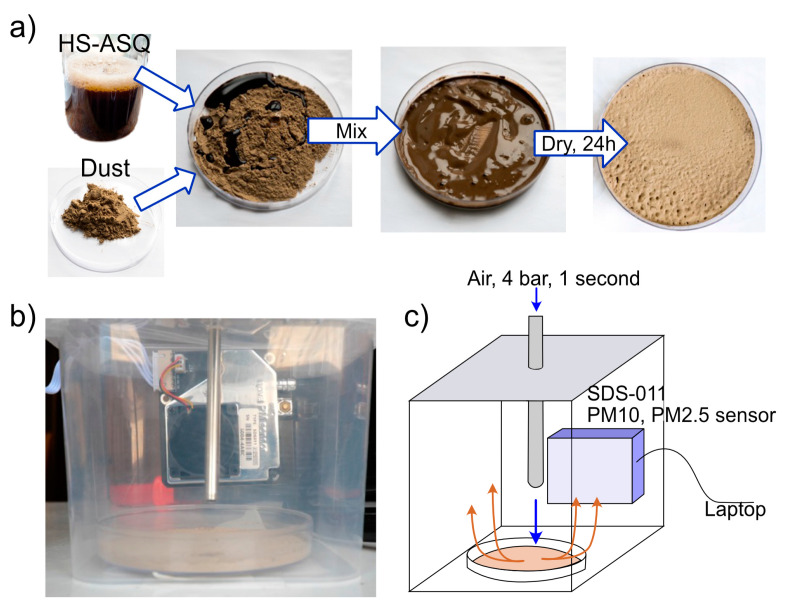
Preparation of beds with samples (**a**) and a photo (**b**) and the scheme (**c**) of the wind erosion resistance test installation.

A dust suppression study was carried out on road dust. For the experiment, 10 g of dust was placed on glass beds (90 mm) and 5 mL of the composition or control solution was added to the dust. The sample was thoroughly mixed and air dried for 24 h (Figure 3a). Three beds were prepared for each sample.

The PM10 and PM2.5 concentrations of the sample beds were measured using a laboratory-scale wind erosion resistant tester, constructed in a similar way to the scheme proposed by Lee et al. [38]. The tester consisted of a sealed container with dimensions of 15 cm × 15 cm × 18 cm, equipped with a compressed air pump and an SDS-011 laser dust sensor (Nova Fitness, Jinan, Shandong, China) operated by an Arduino Uno (Smart Projects, Monza, Italy) for data acquisition. A photo and the installation scheme is presented in Figure 3b,c. For measurements, the sample beds were placed inside the container of the air-blowing tester. A 4 bar air pressure was applied to the sample bed for 1 s through a 5 mm diameter tube positioned 10 cm above the sample. An Arduino-controlled relay controlled the pressure application. Maximum particle content values were recorded 30–60 s after the initiation of air supply.

Statistical data treatment was performed using the SciPy Python package [39] version 1.7.3. A one-way analysis of variance (ANOVA) test was applied to examine the differences between amendment types at a probability level of *p* = 0.05.

## 3. Results and Discussion

### 3.1. Preparation of HS–ASQ and its Aging Process

**Figure 4:**
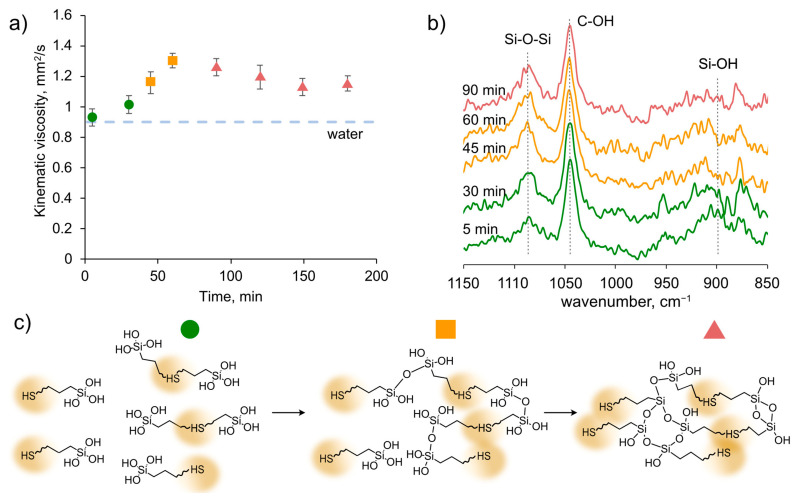
Dependence of kinematic viscosity on time for HS–ASQ:100 5 g/L at 25 °C (**a**), the corresponding IR spectra (**b**), and the HS–ASQ aging scheme (**c**).

For practical use in dust control road treatments, the compositions must be stable and maintain their properties for at least a day. Due to the presence of free silanol groups in HS–ASQ, they are prone to agglomeration by forming cross-linked gels. However, we have previously shown [34] that this process can be controlled by varying the synthetic conditions (pH of the medium, proportion of organosilane, and concentration). In this research, we used pH 8, which enables a moderate rate of silanol group formation and crosslinking. To describe the progress of HS–ASQ ageing, the viscosity was monitored and the IR spectra were analyzed of the HS–ASQ:100 composition. Figure 4 shows the dependence of the kinematic viscosity and FTIR spectra on time, as well as the general scheme of the ageing process.

At the beginning of the reaction, the viscosity was almost equal to the viscosity of water (0.91 mm^2^/s), and after 60 min, the maximum viscosity is reached (1.3 mm^2^/s), then it slowly decreases and reaches a plateau at a value of 1.2 mm^2^/s (Figure 4a). The data obtained can be explained by the following considerations. At the beginning of the process, the system consists of a large number of relatively small individual molecules of humic substances and silsesquioxanes. As a result of the interactions, they grow in size and form larger polyelectrolyte complexes, which assemble into macroaggregates and finally crash out as a separate phase from the water solution. The total concentration of the polyelectrolyte complexes causing a decrease in the viscosity.

Figure 4b shows the FTIR spectra of the corresponding composition at different times. Peaks can be seen in the region of 850–1150 cm^−1^, corresponding to Si-O-Si (1080 cm^−1^), C-OH (1050 cm^−1^), and Si-OH (900 cm^−1^) bonds. The signal of the C-OH bond is mainly due to the ethyl alcohol released during the hydrolysis of APTES; therefore, it was used to normalize the graphs. The spectra are noisy because of the low concentration of HS–ASQ in water, so we cannot draw decisive conclusions. However, there is some increase in the peak intensity in the region of 1080 cm^−1^, and a decrease in the region of 900 cm^−1^, which may correspond to a decrease in silanol groups along with the formation of siloxane bonds. For a more reliable confirmation of ongoing reactions, additional studies are required, which may be the subject of a separate study. The hypothetical process mechanism is shown schematically in Figure 4c.

Thus, the interaction of humic substances with aminoorganosilane results in the formation of polyelectrolyte complexes and their subsequent growth and gel formation. This is why when testing the compositions after synthesis, they were exposed for up to 24 h to achieve stability.

### 3.2. Viscosity Control of HS–ASQ

**Figure 5:**
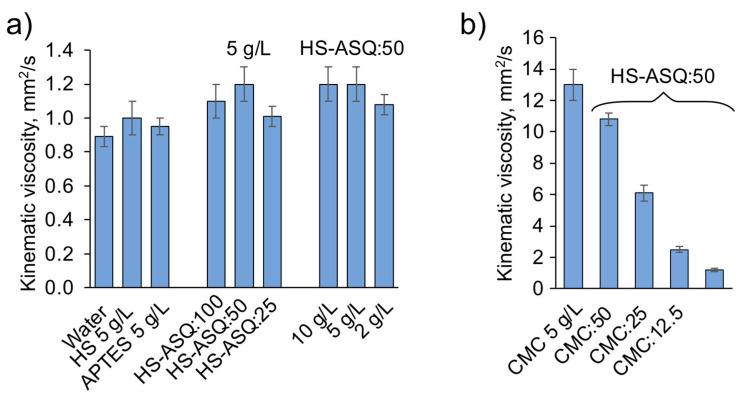
Kinematic viscosity of HS–ASQ compositions and control solutions after 24 h delay (25 °C) without CMC (**a**) and with CMC addition (**b**).

Viscosity is an important parameter of dust suppressors, since it affects both wetting of the dust and the exposition time of the reagents. The viscosity of the dispersion medium plays a crucial role in determining the stability of the solution. A higher viscosity of the dispersion medium results in a greater stability due to its effect on Brownian motion, which slows down the collisions between liquid particles. Polymer materials can also form a robust interfacial layer, which helps to maintain the stability of the system. Thus, a higher solution viscosity leads to improved curing effects. Furthermore, a high concentration of dust suppressant can enhance the cohesion of ground dust, effectively reducing secondary fugitive dust and improving the dust suppression rate [40]. Furthermore, fluid viscosity plays an essential role in spray technology applications, affecting the choice of nozzle type and operating pressure [41]. Therefore, because the viscosities of the HS–ASQ formulations are relatively low, we opted to increase the viscosity with carboxymethylcellulose (CMC) in the study. Due to its low cost, its high ability to swell in water, and its lack of toxicity, CMC is a promising agent for dust suppression, as has been reported in numerous publications [28,38,42]. Figure 5 shows the kinematic viscosities for compositions without (Figure 5a) and with the addition of CMC (Figure 5b).

The viscosity of the HS–ASQ compositions varies from 1 to 1.3 mm^2^/s, and, as can be seen in Figure 5a, it is not significantly affected by a change in the proportion of aminoorganosilane or a change in the concentration in the range of 2–10 g/L. The addition of CMC significantly increases the viscosity of the solutions up to 10.8 mm^2^/s, while phase separation is also not observed, indicating the preservation of active silanol groups. Consequently, with the addition of CMC to HS–ASQ, we can effectively control the viscosity of the composition.

### 3.3. Immobilization of HS–ASQ onto Dust and Morphology Study

**Figure 6:**
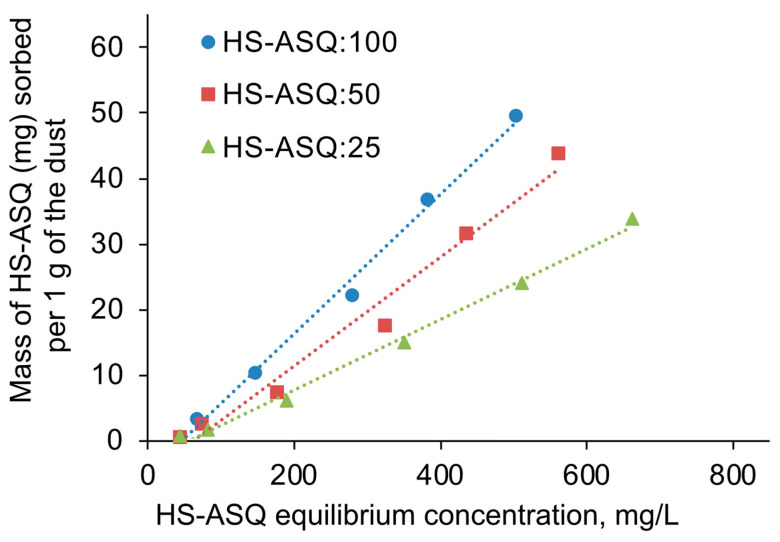
HS–ASQ sorption isotherms onto dust (pH 8.0, 0.1 g of dust per 10 mL of HS–ASQ solution, equilibrium time 24 h, 25 °C).

The isotherm method was used to study the immobilization of silanol derivatives of the HS. To do this, a sample of the dust was added to the HS–ASQ solutions at various concentrations and stirring was carried out for 24 h. Then, the equilibrium concentration and sorption were determined spectrophotometrically. In doing so, for the HS–ASQ:100 composition at initial concentrations greater than 1 g/L, a precipitate occurred after 24 h; therefore, only the initial section of the isotherms, shown in Figure 6, was used to study the sorption process.

The sorption increases with the proportion of organosilane in the composition, which reaches a maximum of 50 mg of complexes per 1 g of dust for the HS–ASQ:100 composition. It can also be noted that, at low adsorbate concentrations, the sorption is almost negligible, and it is necessary to accumulate a certain amount of adsorbate on the surface for effective sorption to begin. This can be explained by the competitive nature of sorption: water is initially sorbed on the dust, and a certain initial concentration of HS silanol derivatives is required to remove it from the surface. Due to the linearity of the studied section of sorption isotherms, they can be described by the Henry equation [43]:

(3) A=K*C,

where *A* is the sorption value, K is the sorption constant, and *C* is the equilibrium concentration.

The calculated sorption constants for compositions HS–ASQ:100, HS–ASQ:50, and HS–ASQ:25 were 0.1063 L/g, 0.083 L/g, and 0.0539 L/g, respectively. This shows a greater sorption capacity of compositions with a greater proportion of organosilane. However, in this case, as was shown earlier, their stability decreases with an increase in the proportion of organosilane in water. Therefore, preparations with a proportion of 25–50% HS are preferable for practical use.

**Figure 7:**
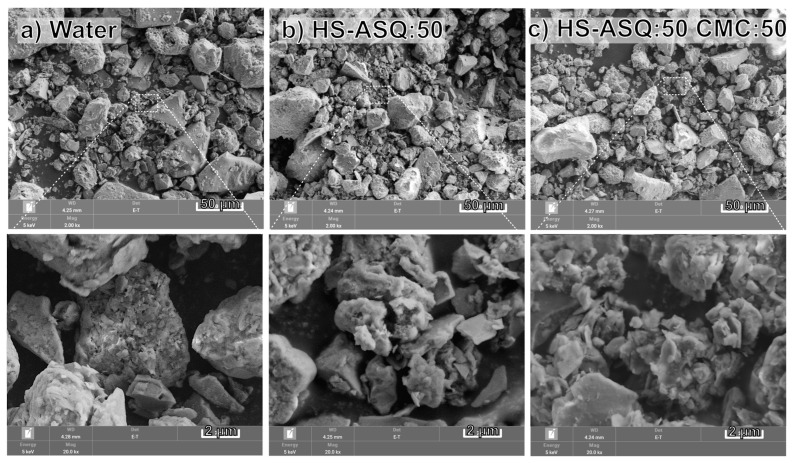
SEM images: surface morphology of dust particles treated with water (**a**), HS–ASQ:50 (**b**), and HS–ASQ:50 CMC:50 (**c**).

Scanning electron microscopy was used to study the morphology of the initial dust, as well as the dust after exposure to HS–ASQ compositions. Figure 7 shows 2000× and 20000× images for slides treated with HS–ASQ:50 and HS–ASQ:50 CMC:50, with water as the control.

Figure 7 shows neither a significant effect on composition nor morphology. All three samples are similar in individual particle sizes, as can be seen from the 2000× images, which show a wide particle size distribution, from small particles of 1–2 µm to large particles of 50–70 µm. However, looking at the 20000× images, it can be seen that the surfaces of the dust treated by the HS–ASQ compositions, especially HS–ASQ:50 CMC:50, are more developed and looser, which may be due to the thin coating of the composition on the surface of the particles. From the data obtained, it follows that the treatment of dust with HS–ASQ compositions does not significantly change the morphology of the particles, and only covers their surface with a film. Therefore, the compositions demonstrate a low cementing force, which may be less effective than some of the more active agents. On the other hand, this leads to a lower environmental impact, which is also essential in the use of dust suppressants.

**Figure 8:**
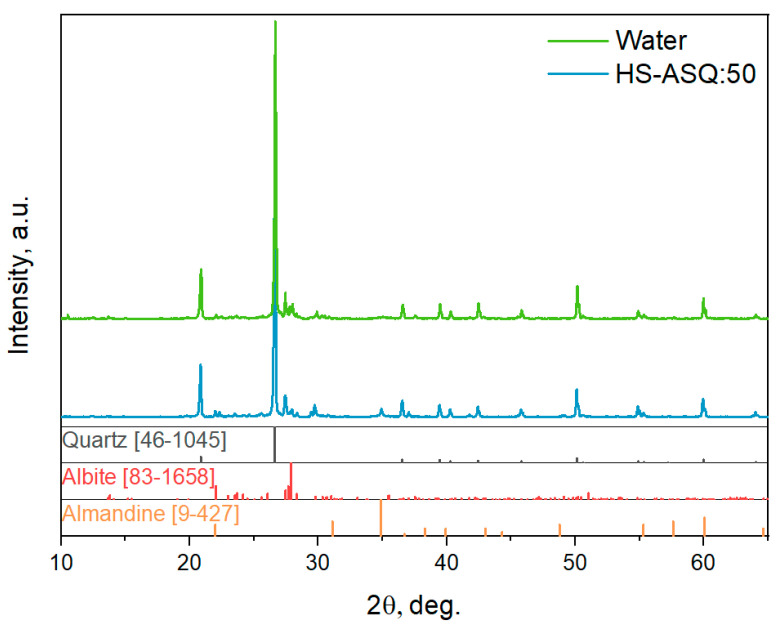
PXRD patterns of dust particles treated with water and with HS–ASQ:50.

Powder X-ray diffraction was used to study the crystalline phase of dust samples. Figure 8 shows the results obtained for the dust sample treated with water and the dust treated with HS–ASQ:50.

Powder X-ray diffraction (Figure 8) demonstrates that the phase compositions of dust treated with water and by the HS–ASQ:50 composition are similar. This can be explained by the small proportion of the introduced preparation relative to the mass of dust, as well as by the X-ray amorphism of humic substances. The main phase presented in the samples is quartz (SiO_2_, ICDD (46-1045)). Additionally, the samples contain minor impurities of iron and aluminum silicate almandine (Fe_3_Al_2_(SiO_4_)_3_, ICDD (9-427)) and mixed potassium and sodium aluminosilicate albite (K_x_Na_1−x_AlSi_3_O_8_, (83-1658)). Thus, the studied dust sample represents particles of a typical mineral origin.

**Figure 9:**
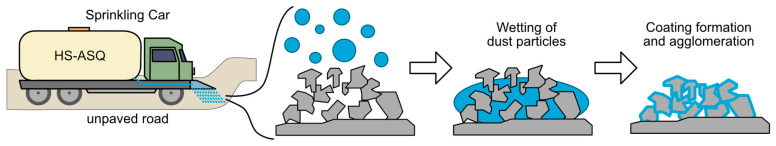
Scheme of the possible mechanism for disposing dust by HS–ASQ.

In the review in [20], the authors considered two possible mechanisms of dust suppression: hygroscopicity and agglomeration. Hygroscopicity is the ability of a substance to absorb moisture from the surrounding atmosphere, preventing dust erosion by keeping the surface damp and compact. Deliquescent substances, which can liquefy if the critical relative humidity is exceeded, are often used for dust suppression. Examples of such agents include salts such as calcium magnesium acetate. Agglomeration-based dust suppression involves adding binding or cementing agents to dust particles to form larger agglomerated particles that are less likely to be airborne. Such agents include corn starch hydrogels, guar gum, chitosan, surfactants, and oil-based substances. We suppose that HS–ASQ combines both of these mechanisms. Figure 9 shows a schematic of the possible mechanisms for disposing dust by HS–ASQ.

In the first stage, dust wetting and the distribution of the composition on the road surface occur. With further drying, a coating is formed on the dust particles, which promotes the binding of particles to each other by the agglomeration mechanism. At the same time, this coating will also have hygroscopic properties due to the incorporation of humic substances, thereby contributing to the long-term retention of water on the road surface, contributing to dust suppression. The presence of humic substances is associated with the water resistant properties of soils [36], where they act according to a similar mechanism. Thus, the use of HS–ASQ will result in nature-like disposal of dust.

### 3.4. Wind Erosion Resistance Test

**Figure 10:**
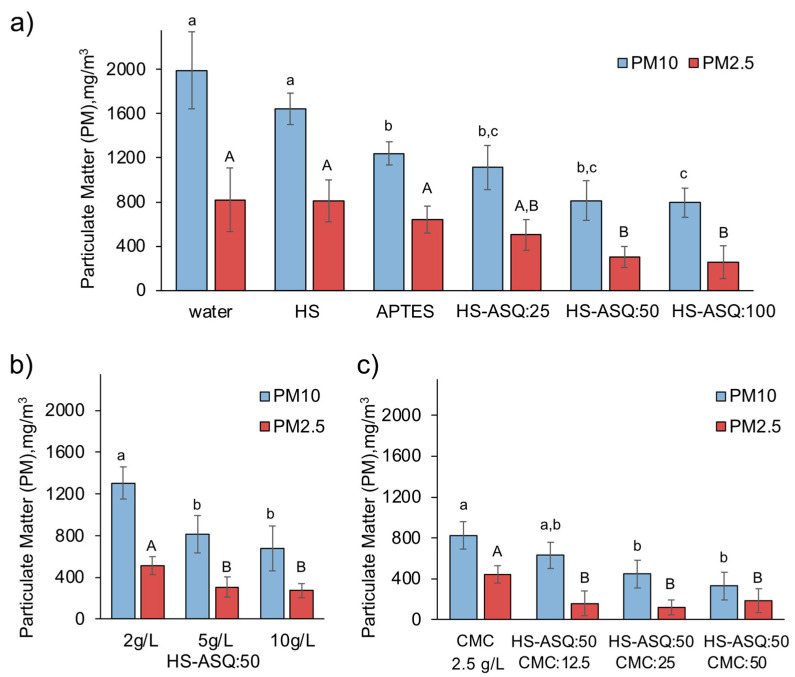
Concentrations of particulate matter (PM10 and PM2.5) in the wind erosion resistance test in the samples treated with various HS–ASQ compositions: control samples and HS–ASQ with different HS:APTES ratios at a concentration of 5 g/L (**a**), with different concentration of HS from 2 g/L to 10 g/L for the HS–ASQ:50 composition (**b**), and with addition of CMC to enhance the viscosity of the HS–ASQ composition at a concentration of 5 g/L (**c**). Values with different letters are significantly different at *p* < 0.05.

A key property of dust suppressors is the resistance to wind erosion. We constructed a laboratory-scale installation to determine this property. A photo and the scheme of the experimental setup are shown in Figure 2. It consists of a plastic box with a laser dust sensor. The sample is placed as a bed and a powerful air jet is switched on for one second, simulating wind. Then, the concentrations of PM10 and PM2.5 are recorded. By comparing the obtained values with those for the control beds, we could judge the effectiveness of the dust suppressors. The test beds were prepared by mixing the HS–ASQ compositions with the dust in a ratio of 1 to 2 by weight and leaving them to dry for one day. We tested a wide range of HS–ASQ compositions with different proportions of organosilane, different concentrations, and with addition of CMC; the test results are presented in Figure 10.

The application of HS–ASQ compositions significantly increased the wind erosion resistance of the treated dust samples when compared to the control (Figure 10a). The control sample treated with water yielded maximum concentrations of particulate matter, at 1990 and 820 mg/m^3^ for PM10 and PM2.5, respectively. At the same time, treatment with humic substances alone or with organosilane alone yielded a negligible effect on the PM2.5 concentration and led to a decrease in the PM10 concentration to 1642 and 1240 mg/m^3^, respectively. Treatment with HS–ASQ compositions resulted in much larger reductions in PM10 and PM2.5 concentrations, from 794 to 255 mg/m^3^, respectively, for compositions with the highest proportion of organosilane, i.e., HS–ASQ:100. The use of compositions with the lower HS:APTES ratio also led to a significant decrease compared to the control, but to a lesser extent; the PM10 concentration decreased to 1113 and 812 mg/m^3^ and the PM2.5 concentration decreased to 503 and 305 mg/m^3^, respectively, for the compositions HS–ASQ:25 and HS–ASQ:50. It can be noted that treatment with HS–ASQ:50 and HS–ASQ:100 led to almost the same effect; therefore, due to the greater stability of HS–ASQ:50, the latter is preferable for practical use. This is why further tests were performed using this composition.

We varied the composition concentrations from 2 to 10 g/L to treat beds with dust (Figure 10b). An increase in the composition concentration from 2 to 5 g/L led to a significant increase in efficiency; the corresponding reduction in the PM10 concentration was from 1305 to 813 mg/m^3^ and for PM 2.5 it reduced from 527 to 305 mg/m^3^. A further increase in the concentration to 10 g/L lead to an additional decrease in PM10 and PM2.5 concentrations to 675 and 272 mg/L, respectively, but the effect was relatively low for a two-fold increase in the composition concentration. This is why it is more reasonable for practical applications to use the HS–ASQ:50 composition at a concentration of 5 g/L.

Carboxymethylcellulose also showed dust suppression capability. Application of CMC yielded values for PM10 and PM2.5 concentrations of 831 and 445 mg/m^3^, respectively, which is significantly lower when compared to the control values. At the same time, a further increase in the dust suppression capability was achieved upon its use together with HS–ASQ compositions. This could be explained by the fact that CMC increases the stability of HS–ASQ compositions and thereby increases its binding to dust particles (Figure 10c). Therefore, when CMC is added to HS–ASQ:50 5 g/l in a proportion of 12.5% relative to humic substances, the concentrations of PM10 and PM2.5 decrease to 630 and 160 mg/m^3^, respectively. Further CMC addition, at 25% and 50%, leads to a further decrease in PM10 and PM2.5 concentrations to 332 mg/m^3^ and 120 mg/m^3^, respectively. At the same time, the effects of 25% and 50% addition are not significant, so 25% of CMC can be considered optimal for use in practice.

## 4. Conclusions

This article explored the possibilities of using humic substance–aminosilsesquioxane complexes as dust suppressors. We have made the following main conclusions:

(1) HS–ASQ is enriched with active silanol groups, the amount of which strongly depends on storage and use conditions. Therefore, it is essential to choose an appropriate HS:APTS ratio and preparation conditions to achieve the properties which will suit the application task. We have shown that the optimum dust suppressing properties are attributable to compositions with an APTES content of 25–50%. In this case, they could be stored and used for more than a day.

(2) Viscosity is another vital property for the proper functioning of binding agents. HS–ASQ solutions have relatively low kinematic viscosity even with high contents of both ingredients (1–1.3 mm^2^/s). This is why the addition of carboxymethylcellulose is advisable, since it allows for a controlled increase in this parameter in HS–ASQ solutions.

(3) HS–ASQ effectively binds to dust particles due to the presence of active silanol groups. The SEM images show that the application of HS–ASQ produces particle aggregates and does not cause an overall cementation of the dust. The formation of particle aggregates mimics this process in soils and has a positive environmental impact.

(4) The best performance in the wind erosion test was demonstrated by the composition consisting of 5 g/L HS–ASQ50:CMC:25. It reduced the concentrations of PM10 and PM2.5 by 77% and 85%, respectively, when compared to the control experiments with distilled water.

The scientific novelty of this work is that for the first time, the possibility of using humic substance–aminosilsesquioxane polyelectrolyte complexes as dust suppressants has been studied. Given the results obtained in the presented study, we can conclude that the humic substance–APTES–CMC compositions have high potential for dust suppression. Of particular importance are the biophilic properties of all constituents, which mimic the natural glue in soil. As a result, the proposed composition and process can be considered as nature-like and its application will not cause harm to the environment.

## Data Availability

The data presented in this study are available on request from the corresponding author.
